# Diagnostic Cut-Off Values Based on Lipid Layer Pattern for Dry Eye Disease Subtypes Assessment

**DOI:** 10.3390/jcm14020623

**Published:** 2025-01-19

**Authors:** Belen Sabucedo-Villamarin, Jacobo Garcia-Queiruga, Hugo Pena-Verdeal, Carlos Garcia-Resua, Eva Yebra-Pimentel, Maria J. Giraldez

**Affiliations:** 1 GI-2092-Optometry, Departamento de Física Aplicada (Área de Optometría), Universidade de Santiago de Compostela, Campus Vida s/n, 15701 Santiago de Compostela, Spain; jacobogarcia.queiruga@usc.es (J.G.-Q.); carlos.garcia.resua@usc.es (C.G.-R.); eva.yebra-pimentel@usc.es (E.Y.-P.); mjesus.giraldez@usc.es (M.J.G.); 2 Instituto de Investigación Sanitaria (IDIS), Travesía da Choupana S/N, 15701 Santiago de Compostela, Spain

**Keywords:** Lipid Layer Pattern (LLP), Aqueous Deficient Dry Eye (ADDE), Evaporative Dry Eye (EDE), Mixed Dry Eye (MDE), cut-off value

## Abstract

**Background**: The aim of the present study was to establish a cut-off value of the Lipid Layer Pattern (LLP) between participants with different subtypes of Dry Eye Disease (DED) including Deficient Dry Eye (ADDE), Evaporative Dry Eye (EDE), and Mixed Dry Eye (MDE). **Methods**: 240 participants diagnosed with DED according to the Tear Film and Ocular Surface Society in the Dry Eye Workshop II guidelines were included in the study. Tear Meniscus Height (TMH) using the Tearscope illumination and Meibomian Gland Loss Area (MGLA) using the Keratograph 5M were assessed to categorize the participants into an ADDE group, EDE group, or MDE group. Then, the LLP was assessed using the Tearscope following the Guillon (LLP-G) and Colour (LLP-C) schemes. **Results**: Receiver Operating Characteristics (ROC) showed that both LLP-G and LLP-C have no diagnostic potential in distinguishing between ADDE and EDE participants (both *p* ≥ 0.724). However, to differentiate the ADDE participants from the MDE, ROC procedures showed a good diagnostic potential with cut-off values of Closed Meshwork-Wave (AUC ± SD = 0.609 ± 0.049, *p* = 0.038, sensitivity: 23.9%; specificity: 76.1%) and Grey-White (AUC ± SD = 0.611 ± 0.050, *p* = 0.034, sensitivity: 40.7%; specificity: 73.9%) for LLP-G and LLP-C, respectively. Also, a significant potential to distinguish between the EDE from MDE participants was found, with cut-off values of Closed Meshwork (AUC ± SD = 0.604 ± 0.049, *p* = 0.043, sensitivity: 40.8%; specificity: 76.1%) and Grey-White (AUC ± SD = 0.604 ± 0.051, *p* = 0.038, sensitivity: 44.7%; specificity: 73.9%) for LLP-G and LLP-C, respectively. **Conclusions**: Using the Tearscope, both LLP-G and LLP-C has diagnostic potential to distinguish MDE participants from the other subtypes of DED.

## 1. Introduction

Dry Eye Disease (DED) is a prevalent condition, affecting 5% to 50% of the global population, posing a challenge for clinicians in their daily routines [1,2]. This condition is characterized by a hyperosmolarity environment, which compromises the homeostasis of the tear film. Consequently, the tear film becomes unstable, resulting in a loss of tear volume, a low break-up time of the tear film and an increased rate of evaporation of the tear from the ocular surface [3]. This is called the “Vicious Circle” of DED [4]. Hence, a correct tear film stability is indispensable in the maintenance of homeostasis and consequently the ocular surface integrity [3,4]. One of the components that plays an essential role in the integrity and upkeep of the tear film is the lipid oil. This lipid oil composes the lipid layer and provides both stabilization and prevention from evaporation [3,5].

On the Tear Film and Ocular Surface Society’s Dry Eye Workshop II (TFOS DEWS II), DED was classified into two main categories: Aqueous Deficient Dry Eye (ADDE), caused by lacrimal gland dysfunction, and Evaporative Dry Eye (EDE), associated with eyelid or meibomian gland abnormalities [6]. However, ADDE and EDE are not two separate entities; they coexist and commonly overlap, contributing to a third type, Mixed Dry Eye (MDE) [4]. MDE occurs when patients have both aqueous tear deficiency and eyelids and/or meibomian glands affected, and it is estimated that about 30% of patients with DED may suffer from this condition [7]. However, it depends on which component is the most affected as to whether it will tend towards a predominantly evaporative or aqueous deficiency [8]. The common methods used to classify DED patients into different subtypes imply the performing of several tests: the assessment of the tear film volume and the status and morphology of meibomian glands, which generates a time burden [8,9,10].

The lipid layer is an essential component of the tear film regardless of DED type [3]. The lipid layer can be assessed by evaluating the Lipid Layer Pattern (LLP) using ocular surface interferometers [5,11]. LLP can estimate the thickness of the tear film lipid layer, which could be a potential tool to differentiate between DED types in a simple way. A thinner LLP is attributed to an EDE and a thicker one to an ADDE [3,11,12]. Currently, correctly identifying a patient’s DED subtype when considering the MDE subtype, in addition to the two main subtypes, can be time-consuming for clinicians because it requires at least two diagnostic tests. This highlights the need to find an easier and single diagnostic test, which simplifies the evaluation, as well as establish a cut-off criterion to distinguish between EDE and MDE or ADDE and MDE, which could establish a categorization rather than a tendency to be one type or the other [4,7]. Therefore, the aim of the present study was to provide a cut-off criterion that strongly discriminates between the DED subtypes including the MDE through the LLP assessment.

## 2. Materials and Methods

### 2.1. Sample

A total of 240 Caucasian participants from the northwest region of Spain were recruited from patients attending the Optometry Service for routine eye examinations. Of these, 185 were women and 55 men, with a mean age of 48.3 ± 16.5 years. The participants were selected based on their compatibility with a DED diagnostic based on TFOS DEWS II criteria. [6]. No one had a prior history of ocular surgery, systemic, or autoimmune diseases, were pregnant or breast-feeding, wore contact lenses or were under medical treatment. Written consent was obtained from all participants, and the study protocol received approval from the institution’s Bioethics Committee (USC-08/2021), ensuring adherence to the principles of the Declaration of Helsinki.

### 2.2. Study Design and Diagnostic Criteria

As outlined in the TFOS DEWS II Diagnostic Methodology report, a series of clinical tests were conducted and documented by a single examiner during one session to minimize interobserver and intersession variability, with measurements subsequently taken by a second blinded observer [11]. The entire study protocol was conducted under controlled environmental conditions, maintaining consistent light, a temperature range of 20–23 °C, and humidity levels between 50–60%.

Procedures were performed from least to most invasive and in the same order for all the participants: Ocular Surface Disease Index (OSDI), tear film osmolarity, Tear Meniscus Height (TMH) with Tearscope illumination LLP, Meibomian Gland Loss Area (MGLA), Fluorescein Break Up Time (FBUT), and corneal staining [6].

**Figure 1:**
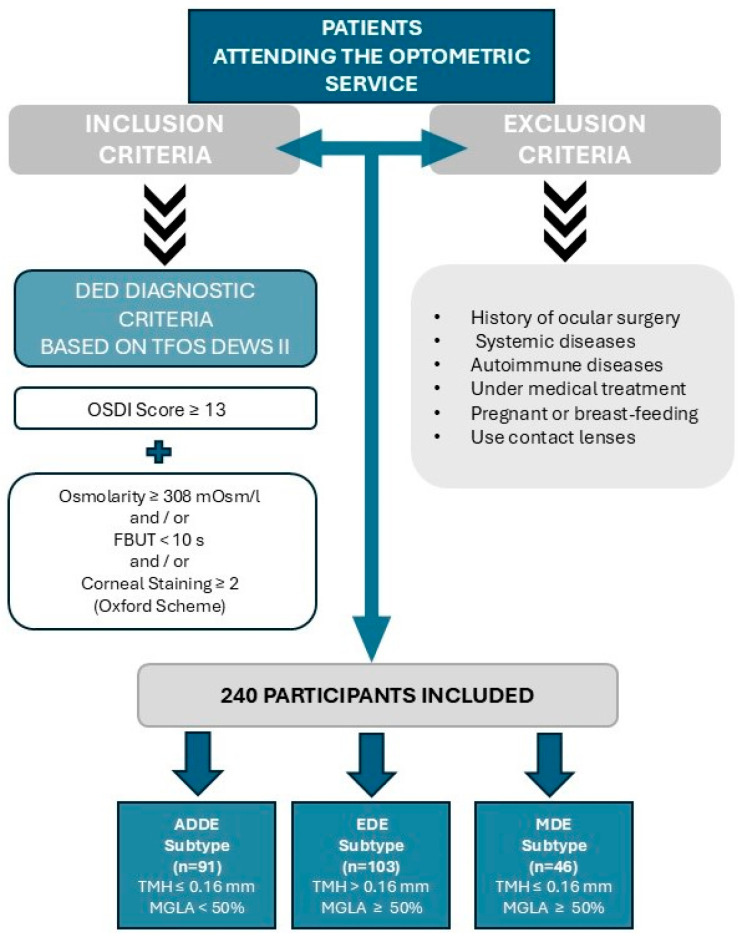
Study design and diagnostic criteria flowchart. DED = Dry Eye Disease; OSDI = Ocular Surface Disease Index; FBUT= Fluorescein Break Up Time; ADDE= Aqueous Deficient Dry Eye; EDE= Evaporative Dry Eye; MDE= Mixed Dry Eye. TMH = Tear Meniscus Height. MGLA= Meibomian Gland Loss Area.

To diagnose DED, the following criteria were used: an OSDI score ≥ 13 combined with at least one of the following signs: tear film osmolarity ≥ 308 mOsm/L, FBUT < 10 s, and/or a corneal staining score ≥ 2 according to the Oxford Scheme (Figure 1) [4,6,13,14]. Once the participants were diagnosed with DED, the sample was divided into three groups following DED subtypes described in the TFOS DEWS II Diagnostic Methodology report [6,9]:

- ADDE subtype: TMH ≤ 0.16 mm and MGLA < 50%.
- EDE subtype: TMH > 0.16 mm and MGLA ≥ 50%.
- MDE subtype: TMH ≤ 0.16 mm and MGLA ≥ 50%.

### 2.3. Evaluation Procedures

#### 2.3.1. Symptomatology Assessment

To quantify the DED symptomatology, the OSDI questionnaire was used, which includes 12 questions for a one-week recall, and was self-administered via a QR code scanned on mobile devices [6,15,16]. Scores, ranging from 0 to 100 points, were assessed by the examiner following standardized guidelines, with higher values indicating greater disability [6,15,16].

#### 2.3.2. Tear Film Osmolarity

Tear film osmolarity was measured using the TearLab osmometer (TearLab Corp, San Diego, CA, USA) [17]. Participants were seated and instructed to look upwards while the device’s probe was carefully positioned on the lower tear meniscus. The examiner allowed the device to emit a beep, signalling that the sample had been successfully collected. [17]. The device translates the electrical impedance of the sample into osmolarity values (mOsm/L) within a range of 275 to 400 mOsm/L, displaying the results on its screen [17]. All measurements were conducted using test cards from the same lot to ensure consistency.

#### 2.3.3. Fluorescein Break-Up Time

FBUT was assessed with the Keratograph 5M (Oculus Optikgerate GmbH, Wetzlar, Germany) and the fluorescein function provided by the device [18,19]. The participants were properly positioned and instructed to look up to the ceiling. Then, a fluorescein strip hydrated with saline was applied to the lower bulbar conjunctiva and participants were instructed to blink several times to ensure an adequate mixing of the dye [20]. Immediately after, they were asked to look straight at a red dot in the device and blink three times to record the FBUT videos. This procedure was repeated three times [9,20]. FBUT was defined as the time interval between the last blink and the appearance of the first dark spot [6,9]. Once the videos were extracted to the computer, the FBUT was assessed using VirtualDub64 v1.10.4, an open software which converts the video recorded into frames (1 s = 8 frames) [9].

#### 2.3.4. Corneal Staining

Ocular surface damage was evaluated through corneal staining measured using the Keratograph 5M, immediately after recording FBUT videos and using the same illumination [18,21]. Participants were instructed to look at a central red dot and perform the four gaze positions while being video recorded [20,22]. After recording and extracting images, corneal staining was assessed using the Oxford Scheme, which grades damage severity from 0 to 5: 0–1 (mild), 2–3 (moderate), and 4–5 (severe).

#### 2.3.5. Tear Meniscus Height

TMH was evaluated using a Tearscope interferometer (Tearscope, Keeler, Windsor, UK) attached to a Topcon SL-D4 slit lamp (Topcon Corporation, Tokyo, Japan) [12]. To standardize the observation area across all videos, the Tearscope was fixed to the slit lamp, maintaining a consistent distance between the chinrest and the device throughout the imaging process. Participants were positioned at the slit lamp, maintaining their primary gaze while blinking naturally to allow observation of the lower tear meniscus. Videos of the meniscus were recorded using a Topcon DC4 camera (Topcon Corporation, Japan) attached to the slit lamp [23,24]. Images were extracted from the recorded videos and analyzed with ImageJ v1.53t software (National Institutes of Health, Bethesda, MD; (http://imagej.nih.gov/ij/ (accessed on 10 October 2024)) [9]. The ImageJ data, initially in pixels, were converted to millimetres for statistical analysis. According to a previous study, 300 pixels equated to 1 mm [9].

#### 2.3.6. Meibomian Gland Loss Area

The visualization of the meibomian glands was performed with the Keratograph 5M. The infrared illumination provided by the device facilitates the observation of the meibomian glands while the lids are everted [10]. The participants were properly positioned onto the device and requested to look up to the ceiling to evert the lower eyelid. Several meibography images were taken and exported from de Keratograph 5M to the computer. Images were extracted from the recorded videos and analyzed with ImageJ v1.53t software (National Institutes of Health, Bethesda, MD; http://imagej.nih.gov/ij/ (accessed on 10 October 2024)) [9]. MGLA categorization followed the Pult et al. [25] scale, featuring four grades: Grade 1 (<25% MGLA), Grade 2 (25–50% MGLA), Grade 3 (50–75% MGLA), and Grade 4 (>75% MGLA).

#### 2.3.7. Lipid Layer Pattern

LLP was also assessed using the Tearscope interferometer [5]. The Tearscope is an interferometer that provides visualization of the LLP of the lipid layer for thickness estimation. Both the device and participants were positioned in the same position as the TMH measurement with their sight straight to the centre of the device, and were instructed to blink three times without squeezing. This process was repeated three times [5,11,26]. The entire process was video recorded. Immediately, LLP images were extracted, at the precise moment when the LLP was stabilized and totally expanded for one second after blinking. Then, LLP images were classified following two different scales.

- First, following the basic Lipid Layer Pattern Guillon’s (LLP-G) scheme in five steps (Open Meshwork, Closed Meshwork, Wave, Amorphous, or Colour) with the intermediate of each as inter-categories [26]. A grade from 1 to 5, with middle steps, was assigned to analyze thickness from thinnest to thickest.
- Secondly, LLP images were classified in four steps following the Lipid Layer Pattern Colour (LLP-C) characteristics scheme (Grey, White, Yellow, Brown or higher) and the intermediate mixtures of colours as inter-categories [27]. A grade from 1 to 4, with middle steps, was assigned to analyze thickness from thinnest to thickest.

In the LLP image classification for both scales, the predominant grade present in the images was chosen. In the absence of this grade, the intermediate grade was chosen.

#### 2.3.8. Statistical Analysis

The data were analyzed using SPSS statistical software version 25.0 for Windows (SPSS Inc., Chicago, IL, USA). The significance level was established at *p* ≤ 0.05 for all statistical tests. Prior to conducting the analysis, an assessment of data normality was conducted using the Kolmogorov–Smirnov test [28,29]. Results indicated that osmolarity and TMH data followed a normal distribution (Kolmogorov–Smirnov, all *p* > 0.05), whereas OSDI, MGLA, corneal staining, FBUT, and both LLP-G and LLP-C did not (Kolmogorov–Smirnov, all *p* < 0.05). Descriptive statistics were calculated using the mean and SD for parametric parameters, and the median with interquartile range (IQR) for non-parametric parameters. The range of minimum and maximum values was reported for both types of data. To assess differences in parameter values between DED subtypes, an ANOVA analysis along with Bonferroni post hoc for paired analyses was used on parametric parameters, whereas Kruskal–Wallis along with the Wilcoxon test for the paired measurement were applied on non-parametric parameters [30]; Bonferroni correction was applied on the Wilcoxon test by adjusting the significance value by the number of comparisons [31].

The study determined the best, both LLP-G and LLP-C, threshold by both classification methods using the Receiver Operating Characteristics (ROCs) analysis to differentiate between participants with different eye conditions [32,33,34]. This process involved evaluating various threshold values and plotting sensitivity against (1-specificity) to determine the optimal threshold. The model’s ability to differentiate conditions was assessed using the Area Under the Curve (AUC) ± SD, with values ranging from 0 (no prediction) to 1 (perfect prediction). Additionally, the 95% Confidence Intervals (CI) for the AUC were calculated (Mean ± 1.96 × SD), and the optimal threshold for each ROC curve was selected using Youden’s J statistic (J = sensitivity + specificity − 1).

To validate the threshold value obtained, a cross-validation analysis was conducted. By using SPSS commands, a sample of 80% of the data was randomly selected, and the LLPs variables were converted into a binary parameter. The association with the initial diagnosis was assessed using Cramer’s V, ranging from 0 (no prediction) to 1 (perfect prediction). The association between this new threshold and the initial diagnosis is based on the TFOS DEWS II Diagnostic Methodology report using Cramer’s V, which ranges from 0 (no predictive ability) to 1 (perfect predictive ability).

## 3. Results

Descriptive statistics for all the measurements of the sample are provided in Table 1, while descriptive statistics for all the measurements on each subgroup are provided in Table 2. The analysis showed that there was no general statistical difference in the osmolarity, FBUT, corneal staining, LLP-G or LLP-C distribution between DED subtype (all *p* ≥ 0.059), whereas a statistical difference was found in the age, OSDI, TMH, and MGLA values (all *p* ≤ 0.001) (Table 2).

### 3.1. Analysis of LLPs Cut-Off Threshold Values to Differentiate ADDE from EDE Participants

**Figure 2:**
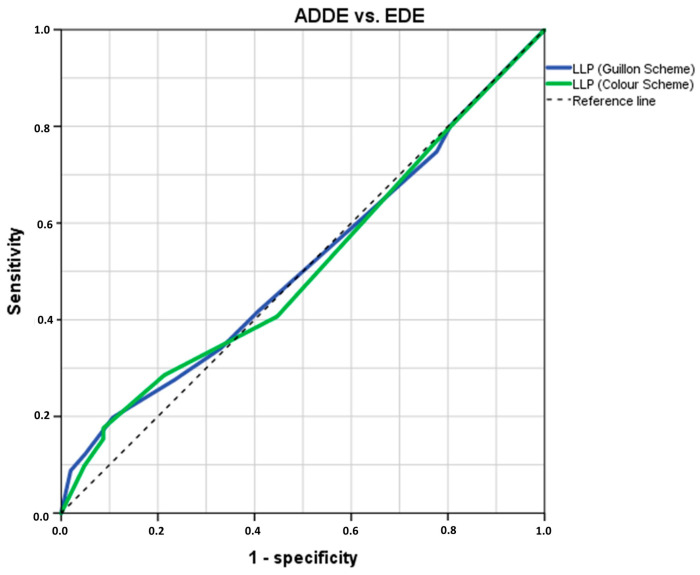
The ROC curve was generated to assess the sensitivity and specificity of the LLP in distinguishing between ADDE and EDE based on theoretical thresholds. The optimal cut-off value was selected at the inflexion point of the curve. n = 194. LLP = Lipid Layer Pattern; ROC = Receiver Operating Characteristic; ADDE = Aqueous Deficiency Dry Eye; EDE = Evaporative Dry Eye.

The pairwise analysis showed that there was no statistical difference in the OSDI, osmolarity, FBUT, corneal staining, LLP-G, or LLP-C distribution between groups (all *p* ≥ 0.376), whereas a statistical difference was found in the age, TMH, and MGLA and values (all *p* ≤ 0.002) (Table 2). The ROC analysis indicated that both LLP-G and LLP-C possesses no diagnostic potential in distinguishing between participant subtypes with an AUC ± SD = 0.515 ± 0.042 (*p* = 0.724, 95% CI = 0.433–0.597) and AUC = 0.509 ± 0.042 (*p* = 0.832, 95% CI = 0.427–0.591), respectively (Figure 2).

### 3.2. Analysis of LLPs Cut-Off Threshold Values to Differentiate ADDE from MDE Participants

The pairwise analysis showed that there was no statistical difference in age, osmolarity, FBUT, corneal staining, or TMH distribution between groups (all *p* ≥ 0.062), whereas a statistical difference was found in the OSDI, MGLA, LLP-G, and LLP-C values (all *p* ≤ 0.032) (Table 2).

**Figure 3:**
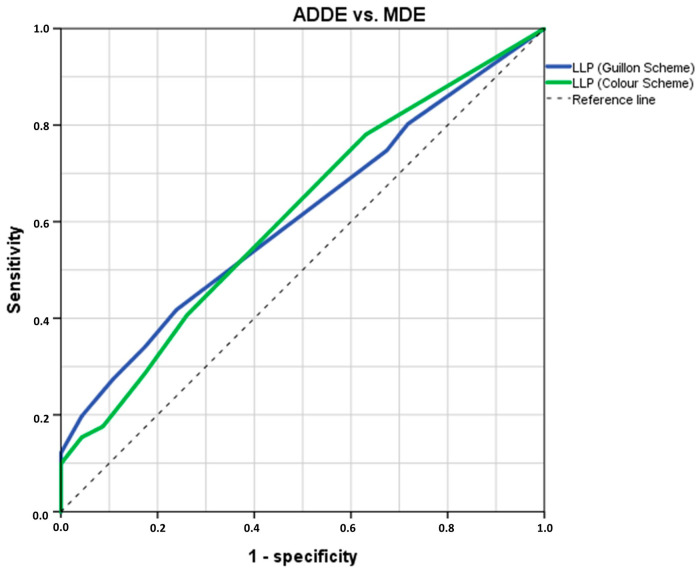
The ROC curve was generated to assess the sensitivity and specificity of the LLP in distinguishing between ADDE and MDE based on theoretical thresholds. The optimal cut-off value was selected at the inflexion point of the curve. n = 137. LLP = Lipid Layer Pattern; ROC = Receiver Operating Characteristic; ADDE = Aqueous Deficiency Dry Eye; MDE = Mixed Dry Eye.

The ROC analysis indicated that both LLP-G and LLP-C possess diagnostic potential in distinguishing between participant types with an AUC ± SD = 0.609 ± 0.049 (*p* = 0.038, 95% CI = 0.513–0.705) and AUC ± SD = 0.611 ± 0.050 (*p* = 0.034, 95% CI = 0.513–0.709), respectively (Figure 3). By computing the Youden’s index for LLP-G (Youden’s J statistic = 0.179) or LLP-C (Youden’s J statistic = 0.146), a cut-off value of Closed Meshwork–Wave (sensitivity: 23.9%; specificity: 76.1%) and Grey-White (sensitivity: 40.7%; specificity: 73.9%) were identified for discriminating between ADDE and MDE participants, respectively. In the cross-validation analysis using an 80% random sample, an association was found between both calculated LLP cut-off values and the previously proposed diagnostic criteria of TFOS DEWS II for distinguishing between ADDE and EDE participants (both, Cramér’s V ≥ 0.175, *p* ≤ 0.041).

### 3.3. Analysis of LLPs Cut-Off Threshold Values to Differentiate EDE from MDE Participants

The pairwise analysis showed that there was no statistical difference in age, osmolarity, FBUT, corneal staining or MGLA distribution between groups (all *p* ≥ 0.757), whereas a statistical difference was found in the OSDI, TMH, LLP-G, and LLP-C values (all *p* ≤ 0.036) (Table 2).

**Figure 4:**
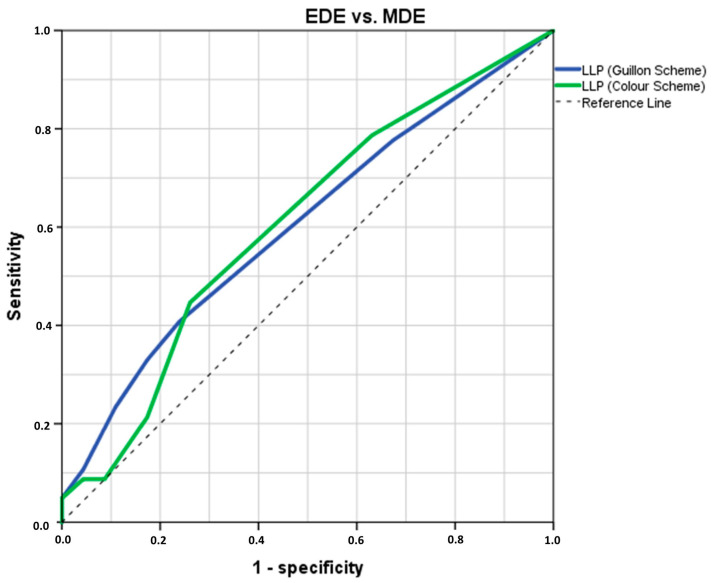
The ROC curve was generated to assess the sensitivity and specificity of the LLP in distinguishing between EDE and MDE based on theoretical thresholds. The optimal cut-off value was selected at the inflexion point of the curve. n = 149. LLP = Lipid Layer Pattern; ROC = Receiver Operating Characteristic; ADDE = Aqueous Deficiency Dry Eye; MDE = Mixed Dry Eye.

The ROC analysis indicated that both LLP-G and LLP-C possesses diagnostic potential in distinguishing between participant types with an AUC ± SD = 0.604 ± 0.049 (*p* = 0.043, 95% CI = 0.508–0.700) and AUC ± SD = 0.604 ± 0.051 (*p* = 0.038, 95% CI = 0.504–0.704), respectively (Figure 3). By computing the Youden’s index for LLP-G (Youden’s J statistic = 0.169) or LLP-C (Youden’s J statistic = 0.617), a cut-off value of Closed Meshwork (sensitivity: 40.8%; specificity: 76.1%) and Grey-White (sensitivity: 44.7%; specificity: 73.9%) were identified for discriminating between EDE and MDE participants, respectively (see Figure 4). In the cross-validation analysis using an 80% random sample, a strong association was found between both calculated LLP cut-off values and the previously proposed diagnostic criteria of TFOS DEWS II for distinguishing between ADDE and EDE participants (both, Cramér’s V ≥ 0.202, *p* ≤ 0.021).

## 4. Discussion

DED is a global condition that represents a challenge in both its management and diagnosis. In this context, the identification of the subtypes has a fundamental relevance [2]. DED has been mainly subdivided into ADDE and EDE subgroups, whereas in the daily practice, this differentiation is not so strict. To distinguish between ADDE from EDE participants, there is a consensus among authors in the use of the TMH or Schirmer as potential diagnosis tests, where cut-off criterions have been stated [6,8,9,35,36]. Also, MGLA has been used to grade the severity of EDE subtype [6,36]. However, there are a significant number of patients who show a combination of signs of both types that are often difficult to differentiate, those known and classified as MDE [1,36,37]. The diagnosis of MDE patients lies in performing the battery of specific tests from both ADDE and EDE subtypes [4,6]. This can be time-consuming and creates the need for simplification to a single diagnostic test. Therefore, the use of the LLP assessment could be useful for establishing cut-off values to differentiate between DED subtypes.

Previous researchers have used the LipiView (J&J Surgical Vision Inc., Irvine, CA, USA) interferometer to measure the lipid layer thickness, and found thinner lipid layers in participants with obstructive meibomian gland disfunction and higher OSDI and SPEED tests values [38,39]. Arita et al. [40] measured the lipid layer thickness with the LipiView interferometer and the LLP with the Kowa DR-1α, reporting that thicker lipid layers were related to the LLP of multicoloured interferometric fringes. Also, Remeseiro et al. [41] stated that lower lipid layer thickness was associated with lower grades on both Guillon’s and Colour schemes. These findings are consistent with those of the present study, specifically because the mean LLP values obtained were within LLP-G from Open Meshwork to Closed Meshwork and within LLP-C from Grey to White, corresponding with the thinnest lipid layers. Also, it should be noted that in the present study, the sample included was entirely composed of DED participants, the EDE subtype being the most prevalent, which explains this trend in the lower LLP grades [4,42]. Additionally, it is important to note that all groups exhibited a similar distribution in terms of the diagnostic criteria of DED. However, significant differences were observed in the classification criteria, where MDE participants had higher OSDI scores, and EDE participants were older than in the other groups.

Chou et al. [38] observed that eyes with thinner lipid layers are more likely to have higher aqueous tear production, and suggested that regardless of the type of DED, the assessment of the LLP could serve as a useful test for DED diagnosis.

The present study found similar results among EDE groups where higher tear production was expected, and showed a Closed Meshwork and Grey-White patterns, representing the thinner lipid layers. On the other hand, participants of the ADDE group that had poor tear production showed thicker lipid layers. These findings encourage the study of the potential diagnostic capability of the LLP; unfortunately, both LLP-G and LLP-C schemes were not able to classify the participants into ADDE or EDE subgroups. This could be due to the fact that the LLP is thinner in EDE (IQR = Closed Meshwork − Wave) but is even thinner in MDE (IQR = Open Meshwork − Closed Meshwork), suggesting that the LLP assessment is not only influenced by lipid production but also by changes in the aqueous layer of the tear film. In EDE, the thinning of the LLP may be mainly due to lipid deficiency, whereas in MDE, both lipid deficiency and changes in the aqueous component contribute to a more pronounced thinning of the LLP. Consequently, the LLP test has difficulty in differentiating between ADDE and EDE as it does not fully capture the complexity of tear film changes in EDE where aqueous influences are less significant. However, the LLP assessment differentiates better between MDE and ADDE and EDE. By setting cut-off values for LLP-G Closed Meshwork-Wave and LLP-C Grey-White, it is possible to differentiate between ADDE and MDE. Similarly, by using the cut-off values for LLP-G Closed Meshwork-Wave and LLP-C Grey-White, it is possible to differentiate between EDE and MDE. The AUC values observed for these were relatively modest, consistent with the previous description of the influence of the aqueous layer on LLP.

Besides the differentiation between ADDE and EDE subtypes from MDE subtype, LLP assessment can also provide information on disease severity when an MDE participant is identified. Since MDE participants have at least a TMH worse than an EDE along with an MGLA greater than an ADDE, the present study showed that the measurement of LLP can estimate the tendency for one of the main DED subtypes of those participants diagnosed as MDE.

The main strength of the study was the large sample size of 240 participants and the provision of a cut-off criterion for LLP assessment to avoid misdiagnosis of participants with MDE. These participants are typically excluded from studies to avoid interference, which can lead to a poorer prognosis. Conversely, the main limitations were the inability of this test to differentiate between ADDE and EDE subtypes, as well as the lower number of participants in the MDE subgroup compared to the other subgroups. In addition, it should be noted that identifying the LLP using different scales requires an experienced examiner. Future studies should validate similar techniques with other interferometers and scales to ensure that this approach is accessible to all clinicians, regardless of their available instrumentation, enabling widespread use of the procedure. As a potential direction for future research, the implementation of this cut-off criterion could play a significant role in the development of an automated system, which could be integrated into multidiagnostic devices. By incorporating this threshold into such devices, it would be possible to streamline the diagnostic process, offering clinicians a more efficient and accurate tool for assessing the severity of dry eye disease. This integration could reduce the need for multiple, time-consuming tests and enhance clinical decision-making, ultimately improving patient outcomes.

## 5. Conclusions

To conclude, the present study proposed an LLP cut-off criterion for Guillon and Colour schemes to differentiate MDE participants from ADDE and EDE participants. This quicker and single procedure not only eliminates the need for an extensive test battery but also enhances the diagnosis of patients with DED. Additionally, it enables more accurate classification of disease severity, ultimately aiding clinicians in providing more effective treatment.

## Figures and Tables

**Table 1:** Descriptive statistics of the entire sample. n = 240.

		Age (Years) *	OSDI (Score) **	Osmolarity (mOsm/L) *	FBUT (s) **	Corneal Staining (Oxford Scheme) **	TMH (mm) *	MGLA **	LLP-G **	LLP-C **
Total Sample (n = 240)	Mean/ Median	48.3	26.04	322.38	5.27	1.00	0.186	53.92	Closed Meshwork	Grey/White
	SD/IQR	16.52	20.12–37.50	18.55	3.43–8.15	0.00–2.00	0.098	41.48–59.67	Open Meshwork/Closed Meshwork—Wave	Grey/White—White
	Minimum	19.0	13.36	282.00	1.29	0.00	0.060	10.24	Open Meshwork	Grey
	Maximum	81.0	83.33	400.00	65.13	4.00	0.640	82.22	Colour	Brown or higher

SD = Standard Deviation. IQR = Interquartile Range. OSDI = Ocular Surface Disease Index. FBUT = Fluorescein Break-Up Time. TMH = Tear Meniscus Height. MGLA = Meibomian Gland Loss Area. LLP-G = Lipid Layer Pattern Guillon Scheme. LLP-C = Lipid Layer Pattern Colour Scheme * Mean and SD displayed on parametric parameters. ** The median and interquartile range (IQR) were used to represent non-parametric parameters.

**Table 2:** Descriptive statistics of the groups.

		Age (Years) *	OSDI (Score) **	Osmolarity (mOsm/l) *	FBUT (s) **	Corneal Staining (Oxford Scheme) **	TMH (mm) *	MGLA **	LLP-G **	LLP-C **
ADDE (n = 91)	Mean/ Median	44.54	25.00	321.47	5.29	1.00	0.128	38.47	Closed Meshwork	Grey/White
	SD/IRQ	17.3	18.75–34.09	17.60	3.42–8.85	0.00–2.00	0.021	28.22–43.51	Open Meshwork/Closed Meshwork—Wave/Amorphous	Grey/White—White/Yellow
	Minimum	19.0	13.36	292.00	1.33	0.00	0.080	10.24	Open Meshwork	Grey
	Maximum	71.0	81.25	400.00	21.83	4.00	0.160	48.78	Colour	Brown or higher
EDE (n = 103)	Mean/ Median	52.7	25.0	322.56	4.79	1.00	0.260	57.02	Closed Meshwork	Grey/White
	SD/IRQ	15.5	20.0–36.36	19.65	3.38–7.38	0.00–2.00	0.110	54.18–62.90	Closed Meshwork—Wave	Grey/White—White
	Minimum	20.0	13.36	282.00	1.29	0.00	0.160	50.38	Open Meshwork	Grey
	Maximum	81.0	83.33	400.00	65.13	4.00	0.640	79.56	Colour	Brown or higher
MDE (n = 46)	Mean/ Median	46.0	35.42	323.78	5.77	1.00	0.129	59.16	Closed Meshwork	Grey/White
	SD/IRQ	15.1	24.43–43.23	18.15	4.02–9.67	0.00–2.00	0.022	55.70–65.01	Open Meshwork—Closed Meshwork	Grey-White
	Minimum	20.0	13.50	284.00	1.75	0.00	0.060	50.38	Open Meshwork	Grey
	Maximum	70.0	75.00	373.00	23.13	4.00	0.160	82.22	Amorphous	Yellow/Brown or higher
	*p*	0.001 ^‡^	0.001 ^†^	0.784 ^‡^	0.190 ^†^	0.655 ^†^	<0.001 ^‡^	<0.001 ^†^	0.066 ^†^	0.059 ^†^

SD = Standard Deviation. IQR = Interquartile Range. OSDI = Ocular Surface Disease Index. FBUT = Fluorescein Break-Up Time. TMH-Tc = Tear Meniscus Height. MGLA = Meibomian Gland Loss Area. LLP-G = Lipid Layer Pattern Guillon Scheme. LLP-C= Lipid Layer Pattern Colour Scheme, ADDE = Aqueous Deficiency Dry Eye, EDE = Evaporative Dry Eye, MDE = Mixed Dry Eye. * Mean and SD displayed on parametric parameters. ** The median and interquartile range (IQR) were used to represent non-parametric parameters. ^‡^ ANOVA for repeated measurements. ^†^ Kruskal–Wallis test.

## Data Availability

Data is unavailable due to privacy restrictions.

## References

[1] Stapleton F., Alves M., Bunya V.Y., Jalbert I., Lekhanont K., Malet F., Na K.S., Schaumberg D., Uchino M., Vehof J. (2017). TFOS DEWS II Epidemiology Report. Ocul. Surf..

[2] Cutrupi F., De Luca A., Di Zazzo A., Micera A., Coassin M., Bonini S. (2023). Real Life Impact of Dry Eye Disease. Semin. Ophthalmol..

[3] Willcox M.D.P., Argueso P., Georgiev G.A., Holopainen J.M., Laurie G.W., Millar T.J., Papas E.B., Rolland J.P., Schmidt T.A., Stahl U. (2017). TFOS DEWS II Tear Film Report. Ocul. Surf..

[4] Craig J.P., Nichols K.K., Akpek E.K., Caffery B., Dua H.S., Joo C.K., Liu Z., Nelson J.D., Nichols J.J., Tsubota K. (2017). TFOS DEWS II Definition and Classification Report. Ocul. Surf..

[5] Guillon J.P. (1998). Abnormal lipid layers. Observation, differential diagnosis, and classification. Adv. Exp. Med. Biol..

[6] Wolffsohn J.S., Arita R., Chalmers R., Djalilian A., Dogru M., Dumbleton K., Gupta P.K., Karpecki P., Lazreg S., Pult H. (2017). TFOS DEWS II Diagnostic Methodology report. Ocul. Surf..

[7] Jones L., Downie L.E., Korb D., Benitez-Del-Castillo J.M., Dana R., Deng S.X., Dong P.N., Geerling G., Hida R.Y., Liu Y. (2017). TFOS DEWS II Management and Therapy Report. Ocul. Surf..

[8] Tsubota K., Yokoi N., Watanabe H., Dogru M., Kojima T., Yamada M., Kinoshita S., Kim H.M., Tchah H.W., Hyon J.Y. (2020). A New Perspective on Dry Eye Classification: Proposal by the Asia Dry Eye Society. Eye Contact Lens.

[9] Sabucedo-Villamarin B., Pena-Verdeal H., Garcia-Queiruga J., Giraldez M.J., Garcia-Resua C., Yebra-Pimentel E. (2022). Categorization of the Aqueous Deficient Dry Eye by a Cut-Off Criterion of TMH Measured with Tearscope. Life.

[10] Fineide F., Arita R., Utheim T.P. (2021). The role of meibography in ocular surface diagnostics: A review. Ocul. Surf..

[11] Garcia-Resua C., Pena-Verdeal H., Minones M., Giraldez M.J., Yebra-Pimentel E. (2014). Interobserver and intraobserver repeatability of lipid layer pattern evaluation by two experienced observers. Contact Lens Anterior Eye J. Br. Contact Lens Assoc..

[12] Guillon J.P. (1998). Use of the Tearscope Plus and attachments in the routine examination of the marginal dry eye contact lens patient. Adv. Exp. Med. Biol..

[13] Garcia-Queiruga J., Pena-Verdeal H., Sabucedo-Villamarin B., Giraldez M.J., Garcia-Resua C., Yebra-Pimentel E. (2023). A cross-sectional study of non-modifiable and modifiable risk factors of dry eye disease states. Contact Lens Anterior Eye J. Br. Contact Lens Assoc..

[14] Sabucedo-Villamarin B., Pena-Verdeal H., Garcia-Queiruga J., Giraldez M.J., Garcia-Resua C., Yebra-Pimentel E. (2023). Longitudinal analysis of variation in status and diagnostic stability of untreated dry eye disease. Ocul. Surf..

[15] Schiffman R.M., Christianson M.D., Jacobsen G., Hirsch J.D., Reis B.L. (2000). Reliability and validity of the Ocular Surface Disease Index. Arch. Ophthalmol..

[16] Miller K.L., Walt J.G., Mink D.R., Satram-Hoang S., Wilson S.E., Perry H.D., Asbell P.A., Pflugfelder S.C. (2010). Minimal clinically important difference for the ocular surface disease index. Arch. Ophthalmol..

[17] Tavakoli A., Markoulli M., Flanagan J., Papas E. (2022). The validity of point of care tear film osmometers in the diagnosis of dry eye. Ophthalmic Physiol. Opt. J. Br. Coll. Ophthalmic Opt..

[18] Zhu K., Xie W., Ying J., Yao Y. (2016). Evaluation of tear film and meibomian gland function in dry eye patients using Keratograph 5M. Zhejiang Da Xue Xue Bao. Yi Xue Ban J. Zhejiang University. Med. Sci..

[19] Itokawa T., Suzuki T., Koh S., Hori Y. (2023). Evaluating the Differences Between Fluorescein Tear Break-up Time and Noninvasive Measurement Techniques. Eye Contact Lens.

[20] Zhao S., Le Q. (2022). Analysis of the first tear film break-up point in Sjogren’s syndrome and non-Sjogren’s syndrome dry eye patients. BMC Ophthalmol..

[21] Inferrera L., Aragona E., Wylegala A., Valastro A., Latino G., Postorino E.I., Gargano R., Orzechowska-Wylegala B., Wylegala E., Roszkowska A.M. (2022). The Role of Hi-Tech Devices in Assessment of Corneal Healing in Patients with Neurotrophic Keratopathy. J. Clin. Med..

[22] Bron A.J., Evans V.E., Smith J.A. (2003). Grading of corneal and conjunctival staining in the context of other dry eye tests. Cornea.

[23] Lam A.K., Tai S.K., Chan J.K., Ng R.W. (2019). Lower Tear Meniscus Height Measurements Using Keratography and Swept-Source Optical Coherence Tomography and Effect of Fluorescein Instillation Methods. Curr. Eye Res..

[24] Garcia-Resua C., Santodomingo-Rubido J., Lira M., Giraldez M.J., Vilar E.Y. (2009). Clinical assessment of the lower tear meniscus height. Ophthalmic Physiol. Opt. J. Br. Coll. Ophthalmic Opt..

[25] Pult H., Riede-Pult B. (2013). Comparison of subjective grading and objective assessment in meibography. Contact Lens Anterior Eye J. Br. Contact Lens Assoc..

[26] Guillon J.P. (1998). Non-invasive Tearscope Plus routine for contact lens fitting. Contact Lens Anterior Eye J. Br. Contact Lens Assoc..

[27] Korb D.R., Baron D.F., Herman J.P., Finnemore V.M., Exford J.M., Hermosa J.L., Leahy C.D., Glonek T., Greiner J.V. (1994). Tear film lipid layer thickness as a function of blinking. Cornea.

[28] Armstrong R.A., Davies L.N., Dunne M.C., Gilmartin B. (2011). Statistical guidelines for clinical studies of human vision. Ophthalmic Physiol. Opt. J. Br. Coll. Ophthalmic Opt..

[29] Armstrong R.A. (2013). Statistical guidelines for the analysis of data obtained from one or both eyes. Ophthalmic Physiol. Opt. J. Br. Coll. Ophthalmic Opt..

[30] Armstrong R.A. (2017). Recommendations for analysis of repeated-measures designs: Testing and correcting for sphericity and use of manova and mixed model analysis. Ophthalmic Physiol. Opt. J. Br. Coll. Ophthalmic Opt..

[31] Armstrong R.A. (2014). When to use the Bonferroni correction. Ophthalmic Physiol. Opt. J. Br. Coll. Ophthalmic Opt..

[32] de Monchy I., Gendron G., Miceli C., Pogorzalek N., Mariette X., Labetoulle M. (2011). Combination of the Schirmer I and phenol red thread tests as a rescue strategy for diagnosis of ocular dryness associated with Sjogren’s syndrome. Investig. Ophthalmol. Vis. Sci..

[33] Hajian-Tilaki K. (2013). Receiver Operating Characteristic (ROC) Curve Analysis for Medical Diagnostic Test Evaluation. Casp. J. Intern. Med..

[34] Nahm F.S. (2022). Receiver operating characteristic curve: Overview and practical use for clinicians. Korean J. Anesthesiol..

[35] Niedernolte B., Trunk L., Wolffsohn J.S., Pult H., Bandlitz S. (2021). Evaluation of tear meniscus height using different clinical methods. Clin. Exp. Optom..

[36] Asiedu K., Dzasimatu S.K., Kyei S. (2019). Clinical subtypes of dry eye in youthful clinical sample in Ghana. Contact Lens Anterior Eye J. Br. Contact Lens Assoc..

[37] Ji Y.W., Seong H., Seo J.G., Park S.Y., Alotaibi M., Choi M., Nam S., Kim T.I., Lee H.K., Seo K.Y. (2022). Evaluation of dry eye subtypes and characteristics using conventional assessments and dynamic tear interferometry. Br. J. Ophthalmol..

[38] Chou Y.B., Fan N.W., Lin P.Y. (2019). Value of lipid layer thickness and blinking pattern in approaching patients with dry eye symptoms. Can. J. Ophthalmol. J. Can. D’ophtalmologie.

[39] Eom Y., Lee J.S., Kang S.Y., Kim H.M., Song J.S. (2013). Correlation between quantitative measurements of tear film lipid layer thickness and meibomian gland loss in patients with obstructive meibomian gland dysfunction and normal controls. Am. J. Ophthalmol..

[40] Arita R., Morishige N., Fujii T., Fukuoka S., Chung J.L., Seo K.Y., Itoh K. (2016). Tear Interferometric Patterns Reflect Clinical Tear Dynamics in Dry Eye Patients. Investig. Ophthalmol. Vis. Sci..

[41] Remeseiro B., Bolon-Canedo V., Peteiro-Barral D., Alonso-Betanzos A., Guijarro-Berdinas B., Mosquera A., Penedo M.G., Sanchez-Marono N. (2014). A methodology for improving tear film lipid layer classification. IEEE J. Biomed. Health Inform..

[42] Rolando M., Merayo-Lloves J. (2022). Management Strategies for Evaporative Dry Eye Disease and Future Perspective. Curr. Eye Res..

